# Anticipatory Race‐Related Stress and General Worry Among Black Women Students at an HBCU: The Mediating Effects of Internal Psychological Resources

**DOI:** 10.1002/cpp.70160

**Published:** 2025-10-29

**Authors:** Tiffany R. Williams, Christy L. Erving, LaShay S. Crayton, Kiara Tookes‐Williams, Reniece Mashburn, Michael A. Moses

**Affiliations:** ^1^ Indiana University School of Medicine Indianapolis Indiana USA; ^2^ Department of Sociology The University of Texas at Austin Austin Texas USA; ^3^ Graduate School of Social Service Fordham University Bronx New York USA; ^4^ Psychological Sciences & Counseling Tennessee State University Nashville Tennessee USA; ^5^ Counseling Psychology, Social Psychology, & Counseling Ball State University Muncie USA

**Keywords:** Black women students, mastery, race‐related stress, resilience, secondary appraisal, worry

## Abstract

The pervasiveness of racism and its impacts on mental health continues to complicate the lives of Black women students at Historically Black Colleges and Universities (HBCUs). Emerging studies also reveal the prevalence of increasing anxiety among Black women. Stress responses culminate in increased mental (e.g., worry), emotional (e.g., shame) and physiological effects (e.g., cortisol reactivity). The biopsychosocial model of racism was used to examine the relationship between anticipatory race‐related stress and general worry among 206 Black women at a HBCU in the southern region of the U.S. Internal psychological factors (secondary appraisal, mastery, resilience and self‐esteem) were evaluated to determine their effects on anticipatory race‐related stress and general worry. Findings indicate a positive relationship between anticipatory race‐related stress and general worry. Further, mastery and self‐esteem partially mediated the relationship between anticipatory race‐related stress and general worry. That is, the association between anticipatory race‐related stress was not as profound when mastery and self‐esteem levels were high. The results highlight the importance of practitioners recognizing the value of internal psychological resources when supporting Black women as they navigate and heal from anticipatory race‐related stress and general worry, thereby reducing potential anxiety.

## Summary

- Anticipatory race‐related stress is related with increased general worry among Black women.
- Independently, secondary appraisal and self‐esteem were the most effective internal psychological factors in reducing general worry.
- Mastery and, to a lesser extent, self‐esteem are strengths that reduce the impact of anticipated race‐related stress on general worry for Black women.
- Practitioners must be mindful of the benefits of internal psychological resources when helping Black women heal from race‐related stress and to cope with general worry.
- Practitioners must exercise cultural humility, sensitivity and competency when working with Black women to promote healing and to avoid further marginalization or racial discrimination.

The pervasiveness of racism and its impacts on mental health continue to negatively impact Black women's lives. As the most common category of mental disorders, Black women disproportionately experience a higher prevalence of anxiety disorders when compared to Black men (Nguyen et al. 2023). Moreover, within their lifetime, 20% of Black women in the U.S. will report symptoms of anxiety (Watson et al. 2012). A growing body of studies is starting to deepen our understanding of how anxiety disorders affect Black women (Hall et al. 2025; Nguyen et al. 2023). A recent study demonstrated a higher prevalence of anxiety among the study sample of Black women than the national average (McCall et al. 2023). Recent research further documents that the highest levels of anxiety are among young Black women (McCall et al. 2023). In fact, generalized anxiety disorder is noted as the most prevalent mental health condition that Black women experience (Lacey et al. 2015). Black women experience greater severity, chronicity and intensity of symptoms than white women (Himle et al. 2009).

Unlike white women, Black women face general life challenges while simultaneously managing a myriad of systemic and oppressive stressors, such as race‐related myths, anticipatory race‐related stress (ARRS), controlling images, stereotypes and adversities that further oppress, marginalize and dominate them (Anonymous Author; Collins 2022; Erving et al. 2022; Lewis et al. 2013; Williams, Erving, Gao, et al. 2023). The uniqueness of the Black woman's experience of stress lies within the intersectional experiences of racism and other oppressions (e.g., sexism). Along with systemic and oppressive stressors, the most common stressors that Black women experience fall into the domains of health, family, relationships, the safety of their children, raising Black children, single parenting, head of household and financial strain (Tipre and Carson 2022). Black women experience greater proportions and burdens of acute and chronic stress leading to greater adverse life outcomes than white women (Kalinowski et al. 2022; Tipre and Carson 2022).

Racism is a stressor that increases Black women's vulnerability to anxiety disorders (Nguyen et al. 2023). The biopsychosocial model of racism (Clark et al. 1999) conceptualizes the experience of racism as a stressor experienced in addition to general stress and depicts it as a stressor that directly impacts well‐being. Black women experience a disproportionate magnitude of social and general stress, and additive generative or accumulation of stress deriving from coping with intersectional oppressions throughout their lifetime and across generations (Hall 2018; Pearlin et al. 1981; Wong et al. 2025). Stress responses culminate in mental (e.g., worry), emotional (e.g., shame) and increased physiological effects (e.g., cortisol reactivity) (Braveman et al. 2017; Johnson 2022).

While emerging studies are shedding light on the prevalence of anxiety among Black women, less is understood about how symptoms of anxiety (e.g., excessive worry, rumination) manifest in this population. The current study contributes to the literature by offering a nuanced investigation of how race‐related stressors and general worry shape the manifestation of anxiety. We must obtain a clearer understanding of the oppressive factors (e.g., ARRS) that contribute to Black women's increased vulnerability to experiencing symptoms of anxiety (e.g., perpetual worry) to shape best practices, develop culturally relevant treatment options and improve mental well‐being.

## 1 What is Racism?

Racism is described as the use of race to subjugate, dominate or assert power over persons of other races with the intent to devalue or deem them inferior (Harrell 2000; Jones 1997). Forms of racism include *individual* or when one asserts racial superiority or dominance over persons of other races (e.g., racial or ethnic microaggressions); *institutional* refers to racial marginalization or oppression depicted through laws, policies, institutions or systems (e.g., “Stop Woke Act”); and *cultural* refers to societal norms, mainstream culture or ideologies (e.g., colonization) that are deeply rooted in the U.S. infrastructure and mainstream culture (Jones 1997; Leath et al. 2025). Black people commonly experience racial or ethnic microaggressions, which are the “brief and commonplace daily verbal, behavioural or environmental indignities, whether intentional or unintentional, that communicate hostile, derogatory or negative racial slights and insults toward people of colour” (Sue et al. 2007, 3). These experiences with racism are commonly subtle and often derive from well‐intentioned individuals. Because of the subtle nature, the recipient of a microaggression may question if a racist event occurred. *Attributional ambiguity* is the act of engaging in *sanity checks* in which the recipient seeks feedback from others to confirm or deny that the situation was racially motivated (Harrell 2000; Sue et al. 2007). Distress often emerges from direct or indirect (vicarious) experiences of racism regardless of overt or subtle exposures. Regardless of the form of racism, Black women experience racial events or anticipated racial events that generate stress in conjunction with general life stress that they experience on a day‐to‐day basis.

## 2 Biopsychosocial Model of Racism

The stress process model was a framework designed to depict the mechanisms through which experiencing stressors results in distress (Pearlin et al. 1981). The stress process model describes the manifestation of stress via causal links between social stressors (e.g., life events, chronic stressors), mediators (e.g., coping, social resources) and symptoms that contribute to health outcomes. Social stressors intersect with and depend on people's social locations, as informed by status hierarchies (e.g., people of low socioeconomic status experiencing economic challenges), thus intensifying adverse mental health symptoms (Aneshensel and Avison 2015). Lazarus and Folkman's (1984) stress‐coping model expanded the original stress process theory with a transactional component in that people interact with their environment, which affects their coping or adaptation capacities (Lazarus and Launier 1978). The biopsychosocial model of racism builds on these stress process theories to describe how racism manifests as a stressor and affects mental and physical health.

The biopsychosocial model of racism (Clark et al. 1999), an extension of broader social stress theories, represents a mechanism by which one can understand how exposure to racism generates stress and negatively impacts one's functioning. Black people experience stress deriving from transactional experiences of racism, racialized violence and other oppressions “that are perceived to tax or exceed existing individual and collective resources or threaten well‐being” (Harrell 2000, 44). The biopsychosocial model of racism postulates that perceptions of race‐related events or other environmental stimuli produce biological, psychological and physiological stress responses (Clark et al. 1999). Additionally, questioning, doubting or challenging that a racism‐related event occurred causes further distress and may fuel accusations suggesting the affected person is paranoid, hostile, overly sensitive, manipulative or intentionally self‐serving (Harrell 2000). Denial or disbelief from others that a racially motivated event occurred or having to provide proof that it happened exacerbates the effects (Harrell 2000).

Race‐related stress produces stress responses that are informed by intersecting systemic, sociocultural, demographic, psychological and behavioural factors and coping resources (Clark et al. 1999; Utsey et al. 2013). Recent investigations on the impacts of racism on Black women include the emergence of mental health outcomes, specifically depression (Quist et al. 1986; Sherman et al. 2023), trauma disorders (Leath et al. 2025; Fani et al. 2021; White et al. 2023), body image (Hughes 2021) and maladaptive eating behaviours (Brown et al. 2022). Racism increases Black women's risk of physical health outcomes (e.g., obesity, hypertension), maternal or pregnancy‐related concerns, life expectancy and infant mortality (Chinn et al. 2021). Clark et al. (1999) proposed several variables deemed to serve as mediators (e.g., adaptive coping responses) or moderators (e.g., socioeconomic status) in the relationship between racism‐related stress and health outcomes. We will discuss more about the mediators under investigation for the current study, but first, we will present the literature on the effects of anticipating race‐related stress.

## 3 Anticipatory Race‐Related Stress and Mental Health

Race‐related stress is a well‐investigated risk factor contributing to poor mental health among Black women (Mekawi et al. 2021, 2022; Odafe et al. 2017). Utsey et al. (2013) provided a psychometrically viable, testable construct, prolonged ARRS, that has four components: *prolonged activation* refers to the extent to which stress lingers beyond initial activation, *perseverative cognition* is the presence of cognitive rumination related to the racism‐related event, secondary appraisal is the evaluation process that determines the resources needed to cope with the anticipation of the stress deriving from a racism‐related event and ARRS refers to the anticipation and stress that a racism‐related event will occur. The focus of the current study was ARRS as a potential predictor of the experience of general worry, a measure that includes common symptoms of anxiety (Startup and Erickson 2006). Secondary appraisal was also of interest to the present investigation and will be discussed in the next section.

Emerging research on ARRS suggests an association with poorer physical and mental health in Black men and women (Erving et al. 2024; Williams et al. 2024). ARRS has been linked to lupus and smoking behaviours among Black women (Spears et al. 2021). With a sample of mostly women, Black professional nurses reported the experience of ARRS despite adapting to historical and structural barriers leading to advancement in the workforce (Byers et al. 2021). Patterson (2021) found an association between perseverative cognition and ARRS and workplace burnout among a sample of Black mental health counsellors. Race‐related stress and perseverative cognition were found to increase Black women's risk of experiencing symptoms of depression (Erving et al. 2024; Watson‐Singleton et al. 2021). Furthermore, ARRS is associated with traumatic stress (Williams, Erving, Gao, et al. 2023). Race‐related stress and ARRS appear to have a direct impact on Black women's mental health.

Few studies have examined the impact of ARRS on the manifestation of anxiety among Black women and even fewer on the experience of general worry. As general worry about experiencing racism is related to chronic stress and predicts anxiety, it is important to investigate the effects of ARRS on worry to improve best practices and treatment of anxiety (Braveman et al. 2017). Black women between the ages of 18 and 34 are reported to be at the highest risk of experiencing anxiety (McCall et al. 2023). Common comorbid conditions that many Black women manage in addition to anxiety include depression, suicidal ideation or posttraumatic stress (Burke et al. 2023; Wright and Lewis 2020). To date, we are aware of only one study that demonstrated an association between racism and increased anxiety mediated through ARRS and dependent on the attentional bias toward threat (e.g., high) in a sample of Black women (Mekawi et al. 2021). Their findings depict the ways anxiety manifests physiologically when deriving from AARS. Mekawi et al. (2021) conducted a study that revealed a nuanced relationship between preservative cognition, depression and attentional bias. Their findings indicated that preservative cognition was linked to depression with a low threat toward attentional bias, which suggests symptoms are modulated based on how their attention is directed. In contexts where attentional bias is not strongly skewed toward threat‐related stimuli, preservative cognition may be directly implicated in the development or maintenance of depression. This highlights the importance of considering the interplay between different cognitive processes when examining the mechanisms underlying mood disorders. The present study builds on this work with an examination of the relationship between the predictor, ARRS, and the outcome, general worry.

Emerging literature suggests that the AARS is related to poor mental health even among Black women attending Historically Black Colleges and Universities (HBCUs; Erving et al. 2024; Williams, Erving, Gao, et al. 2023). As Black people were historically excluded from predominantly white (PWIs) institutions to obtain postsecondary education, HBCUs were originally established to bridge that gap (Njoku and Patton 2017; Shuler et al. 2022). HBCUs continue to be vital for Black students, as evidenced by a 52% enrollment increase in 2022 (Noonan et al. 2013). HBCUs are associated with positive outcomes for Black students, including higher graduation rates and entry into professional roles (Campbell et al. 2019). Black students attending HBCUs report high levels of social support as opposed to their counterparts attending PWIs (Campbell et al. 2019; Himle et al. 2009). Conversely, HBCUs can also present difficulties for Black women, with experiences of misogynoir, limited curricular representation and societal pressures policing their self‐presentation (Grundy 2021; Njoku and Patton 2017; Noonan et al. 2013). Thus, even in one of the most supportive environments, Black women can experience significant stress navigating their educational journey, which is informed by gendered and race‐based discrimination (Blackshear and Hollis 2021; Campbell et al. 2019; Williams, Erving, Gao, et al. 2023). Specifically, sexual, emotional and verbal abuse are common concerns that Black women experience (Hall and Jones 2022). Traumatic stress has been linked to gendered and race‐based discrimination among Black women at HBCUs (Williams, Erving, Frierson, et al. 2023). Moreover, worry and anxiety are linked to experiences of gendered and race‐based discrimination among Black women generally and those attending HBCUs ( Burke et al. 2023; Erving et al. 2022; Williams et al. 2024).

## 4 Internal Psychological Resources That Reduce Stress

Managing the stress generated from racism‐related events requires internal psychological resources. Secondary appraisal, self‐mastery, resilience and self‐esteem are factors identified as having the capacity to reduce stress for Black women. *Secondary appraisal* is a cognitive process that occurs as a person deciphers how to cope with stressful events and determines which coping resources are available to manage the events (Lazarus and Folkman 1984). During this process, pertinent information is evaluated, such as how harmful an event is, the level of coping needed to reduce discomfort, whether there are sufficient resources to cope, previous and similar events that have occurred, and specific actions that can be taken to cope with the stress (Lazarus and Folkman 1984; Utsey et al. 2013). There is evidence suggesting that Black women use secondary appraisal to manage distressing situations. Lewis et al. (2013) found that Black women utilize a secondary appraisal process to aid in cognitive decisions on whether to engage when faced with gendered racial microaggressions. “Picking and choosing one's battles” (Lewis et al. 2013) depicts links among the coping mechanisms, such as resistance, collective and self‐protective, that Black women use in response to experiences of gendered racial microaggressions and to defend against the damaging impacts of these encounters.

Mastery is another psychological resource evidenced to reduce stress among Black women (Jones et al. 2025). *Mastery* refers to an individual's perception of control over their behaviour, thoughts, emotions and livelihood (Keith et al. 2010; Pearlin and Schooler 1978). Like cognitive appraisal, mastery is a process that a person undergoes in assessing stressors, deciphering the harmful effects of the stressors, and determining if the situations can be managed. Mastery has been linked with resilience, coping and positive physical and mental well‐being (Gilster 2016; Jones et al. 2025; Keith et al. 2010; Zanbar et al. 2021). Research also demonstrates that mastery is a protective factor that reduces stress generated by discrimination and promotes wellness for Black women (Erving et al. 2022; Gilster 2016; Jones et al. 2025; Williams et al. 2024).

Resilience is another protective factor that reduces the effects of stress. *Resilience* is defined as a person's capacity to “overcome the negative effects of risk exposure, cope successfully with traumatic experiences and avoid the negative trajectories associated with risks” (Fergus and Zimmerman 2005, 399). In other words, it is one's ability to bounce back after experiencing adversity while continuing to function throughout the struggle. Approaches to measuring resilience include process‐oriented, outcome‐driven or trait‐oriented (Southwick et al. 2014), with trait‐oriented approaches showing more promise and associations with positive psychological effects for Black people (Karaırmak and Figley 2017). Resilience is a personal quality, such as competence, tenacity, spirituality and awareness of control, that aids people in overcoming hardships and achieving positive adjustment and growth, according to the trait‐oriented approach (Hu et al. 2015). Examining resilience is pertinent to understanding Black women's plight in the United States. Resilience is evidenced to reduce the effects of discrimination, minimize psychological distress and improve well‐being among Black women ( Jones et al. 2025; Mushonga et al. 2021; Spates and Slatton 2021; Williams, Erving, Gao, et al. 2023).

Self‐esteem is an internal psychological factor that can serve as a protective factor in reducing stress. *Self‐esteem* refers to how people perceive themselves, their self‐worth and their evaluation of themselves, including their thoughts, emotions and behaviours (Rosenberg 1986). Additionally, people's beliefs, values and ideologies inform their self‐esteem, which has a direct impact on their mental and physical well‐being (Ali et al. 2022; Leath et al. 2025). Self‐esteem aids in building a firm and reliable foundation for overall positive responses to stressors. Previous studies illustrate the role of high self‐esteem on Black women's psychological health and their capacity to regulate emotions productively (Fernandes et al. 2022; Jones et al. 2025; Watson et al. 2016; Williams et al. 2024). For example, the combination of self‐esteem with social support and mastery reduces the effects of gendered racism (Adkins‐Jackson et al. 2023; Erving et al. 2022; Moody and Lewis 2019). Maintaining high self‐esteem aids in strengthening confidence, utilizing healthy coping skills and feeling equipped to handle any challenges or barriers that occur.

## 5 Current Study

With the use of the biopsychosocial model of racism (Clark et al. 1999), the present study evaluates the association between ARRS and general worry among Black women students at a HBCU. As secondary appraisal, mastery, resilience and self‐esteem are evidenced to reduce Black women's experiences of stress, we argue that these internal psychological factors could serve as protective factors against ARRS. The biopsychosocial model of racism suggests mediators that may reduce the impact of racism. We investigated whether secondary appraisal, mastery, resilience and self‐esteem mediate the association between ARRS and general worry. Our hypotheses are the following:

Hypothesis 1 *ARRS will be positively associated with general worry*.

Hypothesis 2 *Secondary appraisal, mastery, resilience and self‐esteem will be negatively associated with general worry*.

Hypothesis 3 *Secondary appraisal, mastery, resilience and self‐esteem will mediate the association between ARRS and general worry*.

## 6 Materials and Methods

### 6.1 Participants and Procedure

The sample comprised 234 Black women enrolled in undergraduate and graduate programs at a southern Historically Black College or University (HBCU). Table 1 reflects the sample demographic details. The current study was part of a larger mixed‐method investigation of Black women's experiences of gendered racism, race‐related stress and their effects on their well‐being. The Institutional Review Board approved the research. All instruments were administered in a survey format online via Qualtrics^XM^ software. Respondents were compensated for their participation by receiving extra credit in their psychology courses via the Sona System or by entering a raffle to win one of six $25 Amazon gift cards. All participants were provided with local mental health resources and the university counselling centre information in the event of any distress experienced during participation in the study.

**TABLE 1:** Descriptive statistics (*N* = 206).

	Mean/Prop.	SD	Min.	Max.
General worry	38.22	13.77	4.00	63.00
Anticipatory race‐related stress	5.53	1.09	1.25	7.00
Secondary appraisal	3.73	1.22	1.00	7.00
Mastery	4.96	1.08	2.00	7.00
Resilience	3.96	0.55	2.00	5.00
Self‐esteem	3.06	0.61	1.10	4.00
Age				
18–22 years (reference)	0.58			
23–30 years	0.19			
31–40 years	0.09			
41 years and older	0.14			
Work status				
Full‐time (reference)	0.34			
Part‐time	0.37			
Not working/other	0.29			
First‐generation college student	0.41			
Sexual minority	0.16			
Marital status				
Single (reference)	0.75			
Married	0.14			
Divorced/separated/widowed/other	0.12			

*Source:* Gendered Racism and Well‐Being Questionnaire, 2020–2021.

## 7 Measures

### 7.1 ARRS

For our independent measure, we used the ARRS subscale from the Prolonged Activation and Anticipatory Race‐Related Stress Scale (PARS; Utsey et al. 2013). PARS is a 17‐item instrument assessing stress appraisal and responses to racism‐related events. PARS begins with an open‐ended item, which asks respondents to reflect on a racism‐related incident that occurred to them or someone close to them. With that incident in mind, respondents were asked to rate a series of items on a 7‐point Likert‐type scale. PARS has four subscales: 1) Perseverative Cognition; 2) Anticipatory Race‐Related Stress Scale‐Psychological; 3) Secondary Appraisal; and 4) Anticipatory Race‐Related Stress Scale‐Bodily Alarm. The first (perseverative cognition) and fourth (ARRS scale‐bodily alarm) subscales were not of theoretical interest, and thus, were not included in this study's analysis.

For the ARRS subscale, respondents were queried about their level of agreement (1 = *strongly disagree* to 7 = *strongly agree*) with four statements: “When I am around white people, I expect them to say or do something racist,” “I believe that most Black people will experience some form of racism in the future,” “I know if I go where there are mostly white people, there is a good chance I will experience racism,” and “I believe there is a good chance that I will experience racism in the future.” Moderate internal consistency was demonstrated for the ARRS subscale at α = 0.70 (Utsey et al. 2013). The internal reliability for the ARRS subscale in our sample was slightly higher at α = 0.81. Convergent validity was reported with significant correlations with the Experience of Discrimination Global and Worry Scales, and Multigroup Ethnic Identity Measure in expected directions (Utsey et al. 2013).

### 7.2 General Worry

To assess general worry as our dependent measure, the Penn State Worry Questionnaire (PSWQ; Meyer et al. 1990) was used. PSWQ is a 16‐item instrument that evaluates one's propensity for excessive and uncontrollable worry. The PSWQ is associated with anxiety disorders, particularly generalized anxiety. Example items included “I never worry about anything” and “I find it easy to dismiss worrisome thoughts”. Participants responded on a 5‐point Likert‐type scale ranging from 0 (*not at all typical of me*) to 4 (*very typical of me*). High internal reliability was reported α = 0.89–0.96 (Meyer et al. 1990; Liu et al. 2022). Internal consistency was high for the current study (α = 0.93). Previous research confirmed convergent and divergent validity (Hopko et al. 2003; Zainal et al. 2021).

### 7.3 Secondary Appraisal

One subscale of the PARS measure was used to evaluate secondary appraisal, which captures the degree to which a person has coping resources to manage racism‐related events (Utsey et al. 2013). The secondary appraisal subscale has four items measured on a 7‐point Likert‐type scale ranging from 1 (*strongly disagree*) to 7 (*strongly agree*). Following the prompt, “Please describe an event/situation involving racism that you or someone close to you (like a family member or close friend) experienced in the past,” participants were asked to respond to each item. Sample items from the secondary appraisal subscale include “At the time the event/situation occurred, I felt prepared to deal with it” and “I felt I had what I needed to deal with the event/situation.” Internal consistency for secondary appraisal was demonstrated as α = 0.80 (Utsey et al. 2013) and the internal reliability for the current study was α = 0.59. The internal reliability for our study is relatively lower compared to the validation study (i.e., Utsey et al. 2013), which could be attributable to differences in sample selection (e.g., Utsey et al. 2013 included women and men), region (e.g., mid‐Atlantic versus U.S. South region) and other unknown sample characteristics. Convergent validity has been illustrated with significant correlations with the Global and Worry subscales of the Experience of Discrimination Scale and the Multigroup Ethnic Identity Measure‐Revised, in expected directions (Utsey et al. 2013).

### 7.4 Mastery

The Pearlin Mastery scale (Pearlin and Schooler 1978) is a 7‐item measure used to examine people's sense of control they feel they have in their lives (Jones et al. 2025). Sample items include “There is little I can do to change many of the important things in my life,” and “Sometimes I feel that I'm being pushed around in life.” Mastery was measured on a 7‐point Likert‐type scale, ranging from 1 (*strongly agree*) to 7 (*strongly disagree*). Items are averaged to produce scores reflective of high levels of mastery. Previous research demonstrates moderate internal consistency α = 0.67–0.77 (Louie 2020; Pearlin and Schooler 1978). Internal reliability for the current study was α = 0.77.

### 7.5 Resilience

The Connor‐Davidson Resilience Scale (CD‐RISC; Connor and Davidson 2003) is a 25‐item unidimensional instrument used to measure psychological resilience or one's capacity to cope with and bounce back from adversity. Sample items included “I am able to adapt to change” and "I can deal with anything that comes my way". Participants were asked to respond to items on a 5‐point Likert scale with responses ranging from 1 (*rarely true*) to 5 (*nearly true all the time*). Scores were summed to produce a total score, and higher scores are indicative of greater resilience. High internal reliability was reported for CD‐RISC with scores ranging from α = 0.91 to α = 0.93, respectively (Brown and Tylka 2011; Castelin and White 2022). Higher scores from totalled responses reflect greater resilience. Internal reliability was good for the current study (α = 0.91).

### 7.6 Self‐Esteem

The Rosenberg Self‐Esteem Scale (RSES; 1986) is a 10‐item instrument that captures people's perceptions and values of themselves. Sample items include “I think I have a number of good qualities,” and “I wish I could have more respect for myself.” Respondents rated items on a 4‐point Likert‐type scale, with response categories ranging from 1 (*strongly agree*) to 4 (*strongly disagree*). A mean score was calculated by averaging each respondent's scores. High internal consistency has been demonstrated in previous research (α = 0.82–0.91) (Chao et al. 2017; Sinclair et al. 2010). The internal reliability for the current study was 0.89. Convergent and divergent validity have also been illustrated for the RSES with significant correlations in expected directions (Sinclair et al. 2010).

### 7.7 Covariates

All models adjusted for several sociodemographic factors: age, work status, first‐generation college student status, sexual minority status, and marital status.

## 8 Data Analysis

We conducted the analysis using STATA 17.0. Approximately 691 individuals started, but only 234 respondents completed the survey. Thus, our sample is only composed of respondents who completed the survey. The sample includes a combination of undergraduate (73%) and graduate (27%) students across various disciplines (e.g., public health, business administration, psychology and engineering). Most of the women were college‐aged (i.e., between 18 and 25 years old; 67%) and identified as a continuing‐generation college student (58%), heterosexual (85%) and single (75%). Though the original study included 234 respondents, 21 were missing data on general worry. There was also missing data for mastery (*N* = 12), self‐esteem (*N* = 8) and resilience (*N* = 4). After conducting listwise deletion, we retained a sample of 206 for the current analysis.

Descriptive statistics (i.e., means, standard deviations, ranges) were reported for all study measures. Pearson correlations were reported for general worry, secondary appraisal, mastery, resilience and self‐esteem, the focal measures for this study. For the multivariable analysis, we treated general worry as a continuous measure; thus, we applied ordinary least squares linear regression. Given the high correlations among some study measures (e.g., psychological resources were highly correlated), we confirmed that multicollinearity was not present in any of the regression models. All models yielded variation inflation factor (VIF) results at values well below 10, the common threshold for identifying problematic multicollinearity in linear regression models (Midi et al. 2010). To test for mediation, we followed the Baron and Kenny (1986) method and tested mediation effects for secondary appraisal, mastery, resilience and self‐esteem.

## 9 Results

Descriptive information on study measures is presented in Table 1. The mean general worry score was 38.22 (standard deviation [SD] = 13.77), a value slightly higher than general college samples but not meeting criteria for clinically significant worry (Startup and Erickson 2006). ARRS was high with a mean score of 5.53 (SD = 1.09). The mean score for secondary appraisal was 3.73 (SD = 1.22). Regarding psychological resources, average mastery (*M* = 4.96; SD = 1.08), resilience (*M* = 3.96; SD = 0.55) and self‐esteem (*M* = 3.06; SD = 0.61) levels were high in this sample. With respect to the study control measures, 58% were between the ages of 18 and 22 years, 19% were between 23 and 30 years, 9% were between 31 and 40 years and 14% were 41 years of age and older. Thirty‐four percent were full‐time workers, 37% worked part‐time and the remaining 29% were either not working or reported some other work status. Forty‐one percent of respondents were first‐generation college students. Sixteen percent were sexual minorities (e.g., identified as lesbian, gay, bisexual). Seventy‐five percent of respondents were single, 14% were married, and 12% were divorced, separated, widowed or had some other non‐specified relationship status.

Pearson correlations among the key study measures are reported in Table 2. There was a positive, significant correlation between ARRS and general worry (r = 0.15, *p* < 0.05). As expected, secondary appraisal was inversely correlated with general worry (r = −0.33, *p* < 0.05). Regarding psychological resources, mastery (r = −0.44, *p* < 0.05), resilience (r = −0.43, *p* < 0.05) and self‐esteem (r = −0.49, *p* < 0.05) were negatively correlated with general worry. In addition to correlations between general worry and other study measures, there was also a significant negative correlation between ARRS and self‐esteem (r = −0.14, *p* < 0.05). It is also noteworthy that the psychological resources were highly correlated with one another, ranging from r = 0.58 to r = 0.67.

**TABLE 2:** Pearson correlations among key study measures (*N* = 206).

	1	2	3	4	5	6
1. General worry	1					
2. Anticipatory race‐related stress	0.15*	1				
3. Secondary appraisal	−0.33*	0.06	1			
4. Mastery	−0.44*	−0.13	0.16*	1		
5. Resilience	−0.43*	−0.01	0.25*	0.58*	1	
6. Self‐esteem	−0.49*	−0.14*	0.21*	0.67*	0.65*	1

* *p* < 0.05.

Table 3 includes unstandardized beta coefficients from the linear regression models assessing the association between ARRS and general worry. In Model 1, ARRS was marginally and positively associated with general worry (β = 1.71, *p* = 0.053), after adjustment for age, work status, first‐generation college student status, sexual minority status and marital status (support for Hypothesis 1). Model 2 adjusted for secondary appraisal, revealing a positive association between ARRS (β = 1.94, *p* < 0.05). In addition, secondary appraisal was negatively associated with general worry (β = − 3.27, *p* < 0.001). Contrary to expectation, secondary appraisal did not reduce the effect of ARRS on general worry. In Model 3, mastery was associated with lower levels of general worry (β = −5.04, *p* < 0.001). The coefficient for ARRS was reduced to 1.09 (*p* = 0.186); when comparing the coefficients for ARRS in Model 1 and Model 3, there was a 36.3% attenuation after adjusting for mastery. Model 4 adjusted for mastery, which was inversely associated with general worry (β = −9.75, *p* < 0.001). In Model 4, the coefficient for ARRS did not change substantially, suggesting that resilience did not reduce the effect of race‐related stress on general worry. Model 5 adjusted for self‐esteem which was associated with lower general worry (β = −10.04, *p* < 0.001). Adjusting for self‐esteem reduced the coefficient for ARRS by 45.0% (comparing Models 1 and 5). Results from Models 2–5 provide strong support for Hypothesis 2 (i.e., all four internal psychological resources were negatively associated with worry).

**TABLE 3:** Unstandardized beta coefficients from OLS Analysis predicting general worry (*N* = 206).

	Model 1	Model 2	Model 3	Model 4	Model 5	Model 6
Anticipatory race‐related stress	1.71^+^	1.94*	1.09	1.66*	0.94	1.21
	(0.88)	(0.84)	(0.82)	(0.81)	(0.80)	(0.78)
Secondary appraisal		−3.27***				−2.34***
		(0.74)				(0.68)
Mastery			−5.04***			−1.94
			(0.87)			(1.07)
Resilience				−9.75***		−2.96
				(1.67)		(2.06)
Self‐esteem					−10.04***	−5.38**
					(1.50)	(2.01)
Constant	28.20***	38.87***	56.93***	68.09***	64.54***	78.48***
	(5.42)	(5.71)	(7.05)	(8.46)	(7.30)	(8.14)
*R* ^2^	0.14	0.22	0.26	0.27	0.30	0.37

*Note:* Standard errors in parentheses. All models adjust for age, work status, first‐generation college student status, sexual minority status and marital status.

* *p* < 0.05.

** *p* < 0.01.

*** *p* < 0.001.

^+^ *p* < 0.10.

In the full model (6; Table 3) inclusive of ARRS, secondary appraisal, mastery, resilience, self‐esteem and all covariates, ARRS was no longer significant (β = 1.21, *p* = 0.122). Model 6 also indicates that 37% of the variation in general worry was explained by key study measures and covariates. Overall, the results presented in Table 3 are suggestive evidence that mastery and self‐esteem may partially mediate the association between ARRS and general worry.

Results from formal mediation analysis using the Baron and Kenny (1986) method are presented in Table 4. Results revealed that mastery mediated 13.56% and self‐esteem mediated 5.03% of the direct effect of ARRS on general worry. Collectively, mastery and self‐esteem mediated 18.59% of the direct effect of ARRS on general worry. Secondary appraisal and resilience were not identified as mediators. Thus, our results yield partial support for Hypothesis 3.

**TABLE 4:** Mediation analysis of secondary appraisal, mastery, resilience and self‐esteem (*N* = 206).

	Average causal mediated effect	Direct effect	Total effect	% of total effect mediated
Secondary appraisal	−0.15	26.50	26.34	0%
Mastery	5.51	−11.93	−6.42	13.56%
Resilience	−0.37	49.06	48.70	0%
Self‐esteem	1.88	20.71	22.59	5.03%

*Note:* Mediation analysis is based on Models 2, 3, 4 and 5 in Table 3. Mediation analysis is based on the Baron and Kenny (1986) method, using the *medeff* command in STATA.

## 10 Discussion

The persistent presence of racism is documented to have detrimental effects on Black women's mental health. For the current study, the biopsychosocial model of racism was used to evaluate the relationship between ARRS and general worry among a sample of Black women attending at HBCUs. The current study expands upon the body of literature demonstrating the effects of ARRS on Black women's quality of life, its relationship with general worry, and the mediating effects of internal psychological factors within HBCU settings. There remains a scarcity of research dedicated solely to exploring the impact of ARRS and the effects of anxiety in Black women's lives; even fewer studies delve into their broader experiences of general worry among Black college‐age women enrolled in HBCUs.

In alignment with our first hypothesis, the current study identified a significant positive relationship between ARRS and general worry, aligning with prior research identifying a linkage between ARRS and depressive symptoms (Erving et al. 2024) as well as trauma symptoms (Williams, Erving, Gao, et al. 2023) among Black women enrolled at HBCUs. These results are also consistent with previous literature that suggests ARRS is associated with mental and physical health conditions (Mekawi et al. 2021; Nguyen et al. 2023; Spears et al. 2021; Watson‐Singleton et al. 2021). Our results further indicate that ARRS elicits general worry irrespective of whether a race‐related event has occurred. In other words, not only does the experience of racism elicit a stress response, but so does the anticipation of a racism‐related event. The mere potential for a racist encounter is perceived as a threat, even in the absence of a direct confrontation (Mekawi et al. 2021; Nguyen et al. 2023; Williams, Erving, Gao, et al. 2023).

When accounting for all four psychological resources, two emerged as especially effective in reducing general worry: secondary appraisal and self‐esteem. Aligning with extant literature, secondary appraisal and self‐esteem are factors that relate to positive mental health effects (Fernandes et al. 2022; Harrell 2000). Likewise, resilience and self‐mastery are internal psychological factors that negatively relate to general worry and may act as protective factors in mitigating worry, anxiety and other mental health conditions (Anonymous Author; Gilster 2016; Jones et al. 2025; Mushonga et al. 2021; Safren and Dale 2018; Spates and Slatton 2021; Zanbar et al. 2021). The findings offer compelling evidence that internal psychological factors can positively impact Black women's mental health, especially those attending HBCUs.

Our findings provided partial support for the hypothesis regarding internal psychological resources and their impact on reducing levels of general worry. When examining the four internal psychological resources in the same regression model, we found that secondary appraisal and self‐esteem especially reduced general worry, which is partially consistent with our second hypothesis. Prior research demonstrates the significance of elevated self‐esteem in contributing to the psychological well‐being of Black women and their ability to effectively regulate their emotions ( Erving et al. 2024; Leath et al. 2025; Watson et al. 2016). In the process of secondary appraisal, relevant factors are assessed, such as the severity of an event, required coping strategies, availability of resources, past experiences and specific actions to manage stress (Lazarus and Folkman 1984; Utsey et al. 2013). There is evidence indicating that Black women utilize secondary appraisal to navigate distressing situations deriving from gendered or race‐based oppression (Lewis et al. 2013). Put simply, individuals who can evaluate situations, employ coping strategies to manage stressors and maintain a positive self‐perception may encounter reduced levels of general worry to a greater extent than having high levels of mastery and resilience within their toolkit of psychosocial resources. When considering these two factors, secondary appraisal and maintaining a positive self‐perception (i.e., high self‐esteem) can contribute to lessening general worry and overall mental well‐being.

Our results provide partial validation for our third hypothesis regarding the mediation of the four internal psychological resources on the association between ARRS and worry. Results from our mediation analysis revealed that mastery and self‐esteem specifically reduced the association between ARRS and worry by 14% and 5%, respectively. Essentially, Black women enrolled at HBCUs who feel more in control of their lives and well‐being and who have high levels of self‐esteem may not experience as much perpetual worry in relation to ARRS.

It is also noteworthy that when all internal psychological factors were entered into the model, the relationship between ARRS and worry was no longer significant. This finding suggests that together internal psychological resources have the potential to substantially reduce the effects of ARRS on general worry. Studies have shown that each of the internal psychological factors independently serves as protective factors that mitigate the stress resulting from discrimination and foster well‐being among Black women (Benson et al. 2025; Williams, Erving, Frierson, et al. 2023; Williams et al. 2024). Each internal psychological resource appears to play a crucial role in establishing a solid and dependable basis for maintaining positive reactions to stressors and in facilitating effective emotion regulation practices (Hall et al. 2025; Watson et al. 2016). Racism is demonstrated to have deleterious effects on Black women's mental health. More research is needed to identify internal strengths and coping resources to facilitate healing and wellness among Black women across multiple contexts and not just HBCUs (Wong et al. 2025).

The results illustrate clinical implications for practitioners. For example, practitioners who help Black women cultivate high levels of mastery and nurture high self‐esteem will not only help improve their sense of self but, as a by‐product, alleviate the psychological impact of ARRS on general worry. Another possible outcome is an overall reduction in vulnerability to anxiety disorders. Practitioners must be mindful of the benefits of internal psychological resources when helping Black women cope and heal from race‐related stress and general worry. Helping Black women capitalize on personal strengths and internal resources will likely reduce stress while improving their overall well‐being. Creating safe, affirming and non‐judgmental spaces in treatment will encourage Black women to share their cultural and racial stories while facilitating critical consciousness, all necessary conditions for achieving an optimal state of wellness and psychological relief. Exercising cultural humility, sensitivity and competency when working with Black women may reduce incidents of further marginalization or unintentionally perpetuating microaggressive incidents. In addition to increased critical awareness of internal psychological resources, Black women may feel empowered to resist racism and internalize healthy coping strategies that prevent general worry from escalating to an anxiety disorder.

## 11 Limitations/Future Directions

Despite the current study's contributions, some limitations should also be considered. First, our measure of ARRS only mentions racism under the condition of white Americans being the sole perpetrators. Nevertheless, Black women (and Black people more generally) experience anti‐Black prejudice at the hands of other racial and ethnic minority groups (Haywood 2017; Tokeshi 2023). Thus, other measures of ARRS could expand to include anticipating instances of anti‐Black racism from other ethnoracial groups. Second, a cross‐sectional analysis was employed, which limits our ability to ascertain the causal direction of the association between ARRS and worry. For instance, there is a possibility that generally anxious individuals might perceive more ARRS. In research on discrimination and mental health, a longitudinal study found that the association between discrimination and psychological distress is bidirectional, suggesting the discriminatory experiences and distress reinforce one another over time (Oates and DeMaris 2022); thus, our findings should be interpreted as a first step in specifying the linkage between anticipating race‐related stress and worry in this population. In addition, our mediation analysis should also be interpreted with caution, as it is unclear whether low levels of mastery or self‐esteem preceded high levels of worry. Future researchers should focus on conducting longitudinal investigations to discern the impacts of ARRS, psychological resources and worry over time. Third, the current study obtained a sample from one Historically Black University; consequently, generalizations are limited to this group. For instance, Black women in college may differ in important ways from those who are older, not currently pursuing higher education or living outside of the U.S. South. Thus, our findings do not generalize to Black women who are not students or Black women living in other parts of the world. More studies within diverse contexts (e.g., predominately white institutions, in the workforce) should be considered for future research. Relatedly, future researchers should examine age diversity among Black women to better understand how race‐related stress impacts worry for different groups of Black women. Moreover, age and developmental stage also play a role in anxiety disorder prevalence (Thomas Tobin et al. 2022) and access to psychosocial resources like mastery and self‐esteem (Brown and Tylka 2011). Moreover, the weathering hypothesis suggests Black women experience accelerated aging with implications for their physical health (Geronimus 1992; Geronimus 2023). Weathering processes might also have implications for psychological well‐being, access to internal psychological resources and race‐related stress experiences. We encourage future research to explore these possibilities.

Fourth, we were only able to examine four internal psychological resources. Future research is necessary to identify more internal psychological resources (e.g., optimism, emotional awareness, regulation), intrapersonal strengths (e.g., self‐compassion, self‐love) and coping strategies (e.g., active coping, spirituality) that can serve as protective factors. As well, culturally relevant evidence‐based treatments must also be investigated to ascertain the capacity to reduce stress generated from racism and worry. Lastly, future research must continue to deepen our understanding of the effects of ARRS on Black women's mental and physical health with more investigations of specific conditions, such as generalized and social anxiety, agoraphobia or obsessive‐compulsive disorder as well as comorbid trauma‐related and mood disorders.

## Ethics Statement

The Tennessee State University Institutional Review Board approved (Protocol #HS‐2020‐4487) this research. The American Psychological Association's Ethical Principles of Psychologists and Code of Conduct guided the development of this manuscript.

## Consent

There were no patients engaged in this work, and thus, consent was not obtained. Informed consent to participate in the study was provided, and electronic consent was obtained.

## Conflicts of Interest

The authors declare no conflicts of interest.
